# Forming and Assessing Student Teams in Software Engineering Courses

**DOI:** 10.1007/978-3-030-58858-8_31

**Published:** 2020-08-18

**Authors:** Henrik Hillestad Løvold, Yngve Lindsjørn, Viktoria Stray

**Affiliations:** 6 IT University of Copenhagen, Copenhagen, Denmark; 7 University of British Columbia, Vancouver, BC Canada; Department of Informatics, University of Oslo, Oslo, Norway

## Abstract

In software development projects, working in teams is essential. Therefore, software engineering courses often require the students to be working in teams to learn about team work behaviors and practices. The instructors of software engineering courses are presented with several challenges when teaching courses that require teamwork. For example, how to form high-performing student teams, and how to assess their work. The aim of this study is to evaluate whether there are differences in performance whether the students form the teams themselves, or if the teams are formed by the instructor. We evaluated a course involving agile software development by 200 students working in 39 teams. A total of 76% of the students chose to form their own teams, the remaining 24% were placed in teams by the instructors. Our findings indicate that teams formed by the students perform slightly better than the teams formed by the instructors.

## Introduction

To better prepare software engineering students for real work-life, it is important to let them experience developing software in project teams. A main goal with teamwork is that the participants value working together and learning from each other. Teamwork in software engineering projects is harder than the students expect [1]. A common problem with teamwork is a lack of commitment and contribution of one or more members of the team [4] and communication challenges among the team members [3]. Therefore, understanding how to form teams that experience successful teamwork where everyone learns and contributes is of vital importance.

There are many ways to form teams, ranging from the simple randomizing of teams, to hand-picking students for each team based on their qualifications. Research within the field of formation of teams (also called team composition [3] and group selection [2]) within software engineering courses at the undergraduate level is scarce. Some research on team formation within software engineering in general has been carried out in the US, pointing instructors towards forming teams themselves, without the involvement of the students [9]. Other research points to using algorithm-based tools to automatically match students [5], or by using personality tests to match team members [8].

Oakley et al. [9] found that simply putting students in groups to work on assignments is not a sufficient condition for achieving the benefits of cooperative learning and working in teams. One of the findings in this study is that the teams should establish policies that will govern their operation and get them to formulate their own expectations of one another using a *Team Policies Statement and the Team Expectations Agreement*.

There seems to be no consensus on which way actually leads to more learning and better results in terms of students’ overall performance. Motivated by this, we aimed to investigate the topic of team formation in a large software engineering course.

## Methods

In the spring of 2019, the University offered a software engineering course involving a major agile project where the students worked in teams to develop a mobile application. The course was 20 ECTS credits; equivalent to a total workload of 33% of one full academic year in Norway. The course was made mandatory for second-year undergraduate students following three study programmes; *Programming and Systems Architecture (ProSA)*, *Design, Use and Interaction (Design)* and *Digital Economy and Leadership (DigØk)*. This study was carried out using the data recorded from the teams participating in this course.

### Course Design

During a project period of 13 weeks, students were assigned to write an app for the Android operating system involving API data gathered from the Institute of Meteorology. The students were given introductions to agile methods of software engineering, Scrum and Kanban in particular. All work was to be logged and end up in a report which was then assessed together with the final product and scored on a scale of 0–50 points. The report and product were assessed using the criteria presented in Table 1.

In our course, all the teams followed an agile project model. While Scrum was the process model most focused on in the lectures, this was not the most used process model among the teams. Scrum was chosen by 17 teams. However, the majority of the teams incorporated Kanban elements into their Scrum process models. This process model, ScrumBan, was chosen by 21 teams. The two most popular tools to use in the teams were Trello (used by 24 teams) and Slack (used by 20 teams). Trello was used to keep track of tasks and visualize the workflow. Slack was used to communicate and coordinate, and this tool has been shown to increase team awareness and communication in agile teams [10]. In our teaching, we aimed to focus both technical and soft skills. This applies in the learning elements of the course such as lectures, weekly tasks and mandatory assignments, as well as in the assessment of the report and product as seen in Table 1.

**Table 1.** Criteria for the evaluation of student reports and products, and their percentage of total score.

Criteria	% of score
Title, abstract, team presentation, introduction	4
User documentation	11
Requirements analysis, modelling, patterns	15
Technical product documentation	15
Testing and test documentation	8
Process documentation, reflection on process	19
Overall impression, language, context	12
References, sources, appendices	4
Product and functionality	12

The report and product accounted for 50% of the final grade given to students, the other half being the result of a final individual written exam. The questions on the exam were both from theory presented in lectures and group sessions and from the project they were a part of in the teamwork.

As we can see from Table 1, we assessed the product and functionality of the projects. This includes the source code written by the teams; an aspect which is inherently difficult to assess. In many courses where students write code, only the final outcome and the product is assessed. We found it important not only to look at the outcome and product, but also the source code, as this gives us better insight in the architecture and design patterns chosen by the students, and how this is reflected on in their final report.

### Forming Teams

Early on in the process of designing the course, the question about how teams were to be formed, and how involved in the forming of teams the instructors were to be, arose. Initially, we aimed to minimize the work required by the lecturers, and wanted all students to form their own teams consisting of 4–6 students. We quickly became concerned about the students forming too homogeneous teams in terms of study programme, gender and workload capacity. We were also concerned that students who did not have a social network at the campus would fall behind and not find other students to work together with. To solve these problems, we went with a middle-ground solution where students could choose to either form teams on their own, or be placed in a group manually by the lecturers, based on the following: study programme, ambitions, and availability.

We initially aimed to make the teams as diverse as possible with regards to study programme and gender, whilst minimising the distance between group members level of ambition. This is in line with previous studies within the field with successful results [11]. The students were also instructed to report to the instructors immediately if any signs of dysfunction occurred. This would then lead to a conversation with the course administration in order to solve the problems as they arose.

## Results

In total, 76.3% of the students opted to organise teams by themselves, without the involvement of the instructor. The rest of the students who answered wanted to be placed in teams by the instructor. We were not surprised that the students opting to be placed in teams by the instructor were outnumbered by students opting to form teams on their own; these are second-year students who know each other well and many have already formed study groups.

Unsurprisingly, most students (68.7%) answered that their ambitions were to aim for grades A-B. About a third answered (31.3%) that they aimed at an average grade, and no students answered that they were happy about just passing the course. Furthermore, it is interesting to note that no students were happy as long as they passed.

### Group Formation Outcome

The instructors assessed the results of the survey and put together nine teams of five to six individuals. Six of the teams were within the A-B ambition level, and three of the teams were in the average grade ambition level.

**Table 2.** Gender distribution of teams formed by the instructors and teams formed by the students. M denotes male, F denotes female.

	M only	F only	One F	Mixed
Instructor formed	3	0	0	6
Student formed	9	2	5	14

As we see from Table 2, three teams formed by the instructors consisted of only males, and males were over-represented in all but one team formed by the instructor. For the 9 teams formed by the instructors 27% of the students were female. For the 30 teams formed by the students themselves 31% of the students were female. While some research suggests the gender balance within the team is irrelevant in regards to result [6], we wanted our teams to be diverse. We made it a rule that teams formed by the instructors should at least have two students of each gender, or otherwise be a single-gender team. This was to prevent one student from becoming the “odd one out”, and thus purely for social reasons. However, in the student-formed teams, five of the teams chose to have only one female. The average team size across all teams was 5.21.

**Fig. 1.**
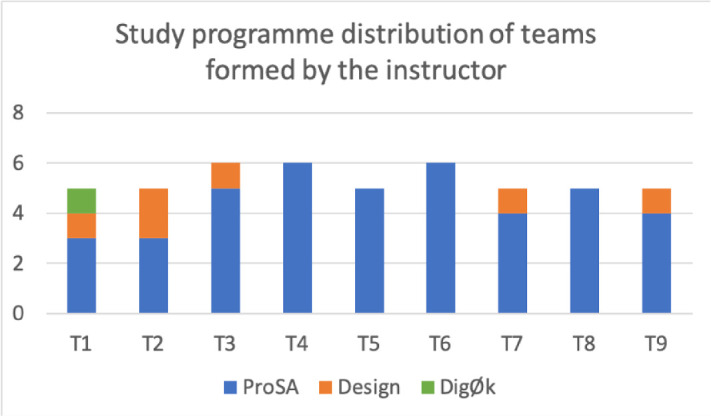
Distribution of students from each study programme grouped by team, from the teams formed by the instructors.

**Fig. 2.**
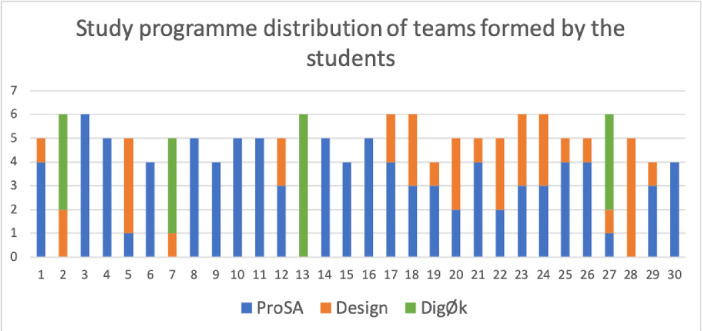
Distribution of students from each study programme grouped by team, from the teams formed by the students themselves.

As for study programme, on the other hand, we wanted diversity. Mishra et al. state that most of the tasks of software development organizations are diverse in nature, and suggests that Software Engineering educators should seek diversity when preparing students for the industry [7]. Figure 1 shows the distribution of students with regards to study programme, grouped by teams. We can read from the figure that students from the programme *ProSA* were over-represented. This comes as no surprise as this by far is the largest study programme at the department with regards to number of students.

For the self organised teams, as we can read from Fig. 2, there were 99 from *ProSA*, 37 from *Design* and 17 from *DigØk*. 12 of the 30 teams had team members from a single study programme, 10 of them were from *ProSA*, 1 from *Design*, and 1 from *DigØk*. It is interesting to note that the student-made teams seem to be just as diverse as the teams formed by the instructors in terms of study programme. This is likely a result of the students signing up to be placed in groups by the instructors mainly coming from a single study programme (ProSA).

### Project Performance

The first section of Table 3 shows the average final points on a scale between 0 and 50 for all teams, grouped by those formed by students and those formed by the instructor, as well as the standard deviation within the teams. As we can see from the results, the teams formed by the students themselves performed slightly better than the teams formed by the instructor. The difference is, however, well within one standard deviation, and with a p-value of $p = 0,114$ we cannot draw a clear conclusion from our data.

**Table 3.** Team project score and individual exam score grouped by student-formed and instructor-formed teams.

	Average points	Standard deviation
*Team score*		
Student-formed teams	42.5	4.70
Instructor-formed teams	40.3	4.27
*Individual exam score*		
Student-formed teams	40.1	6.47
Instructor-formed teams	38.5	7.55

Although the data is somewhat inconclusive, it is interesting to note that the results seem to indicate that teams formed by the students themselves perform slightly better than teams formed by the instructor. The implications of these results will be further analysed in Sect. 4.

### Individual Exam

In addition to the project report and the software product, all students had an individual exam with questions from the curriculum and from the project and teamwork. The second section of Table 3 shows the average points for the individual exam on a scale from 0 to 50 for all teams, grouped by those formed by students and those formed by the instructor, as well as the standard deviation within the teams. For each team we calculated the average of the points (exam results) for all the individual team members in the team. The results are similar to the results presented in Table 3 for average team score, but with a higher standard deviation due to the differences in the results of the individual team members within the teams.

## Discussion

In this study we have analysed the results of student teams in a large 20 ECTS course on software engineering. The students were given the choice to either form teams on their own, or be placed in a team by the instructors based on a small questionnaire at the beginning of the semester. The instructors’ goal was to make teams as diverse as possible, as previous studies seem to support the claim that diverse teams perform better overall than homogeneous teams [11].

We found that 31% of the students chose to be placed in teams by the instructor, while the majority (69%) formed their own teams. Many of the students who opted to form their own teams probably knew each other well on beforehand. This course was offered exclusively to students in their 4th semester of computer science studies, and it is not unlikely that many of the students already had a group of 4–6 peers with which they have collaborated with on other courses. This means that the students who formed teams on their own had the advantage of already knowing they work well together with their teammates, compared to the students who were placed in teams by the instructor.

Furthermore, our analysis might indicate that individuals in teams formed by the students themselves performed slightly better than the individuals in teams formed by the instructors, both on the team evaluation, and the individual final exam.

### Study Limitations

Although we aimed to make the teams as diverse as possible, we do not know the level of diversity, both professionally and in terms of gender and study programme of the teams that the students created themselves. In other words; we cannot be sure whether the teams created by the instructor are, in fact, more diverse than the teams created by the students themselves. We did, though, find it plausible to assume that the general level of diversity probably was in fact lower, as students from the same programmes usually attend the same lectures and spend spare time together.

Although more than 200 students attended the course, and with 39 teams included in our study, there were only 9 teams formed by the instructors. A sample size this small makes it hard to draw definite conclusions, as personal factors of each student within the teams affect the final result.

## Conclusion and Future Work

We studied 39 teams in a major software engineering course. Our results suggest that teams formed by the instructors intended to be as diverse as possible, do not necessarily perform better than teams formed by the students themselves. Our data indicate that the teams formed by the students perform slightly better, but there is no significant difference between the teams in each group. We have no conclusive evidence of why this is the case, but we assume that the social factor plays a major role in this regard. Teams consisting of peers who know each other on beforehand have an advantage over teams who have to get to know each other before starting to work.

Future research should go deeper into investigating if and why student-formed groups perform better, as well as to analyse the effect of diversity. Furthermore, it is a need to understand how this diversity affects the quality of the different parts of the project (such as testing, documentation, usability and maintainability). It would also be interesting to investigate how teamwork quality, meeting frequency and agile practices differed with regards to how the teams were formed. As other research shows promising results using algorithm-based tools to match students [5], this might also be something worth looking further into.

This course was offered again in spring 2020 with a different approach to forming teams. Students could not select all team members by themselves, but were instructed to suggest 1–3 peers they wanted to have in their team. Based on their wishes, we put together teams of 5–6 members, and thus all students had to work with at least one team member they did not know beforehand. This approach has shown promising results, which might be worth looking further into.
